# The Impact of Delayed Symptomatic Treatment Implementation in the Intensive Care Unit

**DOI:** 10.3390/healthcare10010035

**Published:** 2021-12-25

**Authors:** Lesley Meng, Krzysztof Laudanski, Mariana Restrepo, Ann Huffenberger, Christian Terwiesch

**Affiliations:** 1 Yale School of Management, Yale University, New Haven, CT 06511, USA; 2 The Department of Anesthesiology and Critical Care Medicine, Department of Neurology, The Leonard Davis Institute of Health Economics, University of Pennsylvania, Philadelphia, PA 19104, USA; klaudanski@gmail.com; 3 College of Arts and Sciences, University of Pennsylvania, Philadelphia, PA 19104, USA; rmariana@sas.upenn.edu; 4 The Perelman School of Medicine, University of Pennsylvania, Philadelphia, PA 19104, USA; Ann.Huffenberger@uphs.upenn.edu; 5 The Wharton School, The Perelman School of Medicine, The Leonard Davis Institute of Health Economics, University of Pennsylvania, Philadelphia, PA 19104, USA; terwiesch@wharton.upenn.edu

**Keywords:** critical care patient management, intensive care unit operations, medication delay, patient health measurement, quality of health care, empirical analysis, instrumental variables

## Abstract

We estimated the harm related to medication delivery delays across 12,474 medication administration instances in an intensive care unit using retrospective data in a large urban academic medical center between 2012 and 2015. We leveraged an instrumental variables (IV) approach that addresses unobserved confounds in this setting. We focused on nurse shift changes as disruptors of timely medication (vasodilators, antipyretics, and bronchodilators) delivery to estimate the impact of delay. The average delay around a nurse shift change was 60.8 min (*p* < 0.001) for antipyretics, 39.5 min (*p* < 0.001) for bronchodilators, and 57.1 min (*p* < 0.001) for vasodilators. This delay can increase the odds of developing a fever by 32.94%, tachypnea by 79.5%, and hypertension by 134%, respectively. Compared to estimates generated by a naïve regression approach, our IV estimates tend to be higher, suggesting the existence of a bias from providers prioritizing more critical patients.

## 1. Introduction

Over the past three decades, the timely delivery of care has been among the highest priorities as a growing body of evidence has determined that inappropriate care delivery results in increased mortality and morbidity [1,2,3,4,5]. In emergency medicine, the negative impact of prolonged ambulance travel times and longer than usual waiting times for emergency surgery adversely affect care delivery and medical outcomes [6,7,8,9]. Waiting times for primary care appointments show similar trends [10]. Delays in access to care are potentially behind the rapid decline of patients’ mental health [11]. Delays in inpatient physician visits due to off-service placement result in a longer length of stay [12]. This strongly suggests that delays are uniformly linked to negative outcomes in medicine when treatment is protocolized, obligatory, and supported by robust evidentiary support [13,14,15].

Due to the unique dynamics of patient conditions and the elevated acuity of their illnesses, critical care patients require almost continuous monitoring and frequent therapeutic interventions [16,17,18]. Studies have looked into medication delivery gaps in intensive care unit (ICU) settings [19,20,21]. Delays in the transfer from the emergency department to the ICU have been shown to result in a longer ICU length of stay, but this can be attributed to a lack of adequate staffing, among a myriad of other factors [5,22]. The increased emphasis on sepsis cases in the recent literature has demonstrated that delays in antibiotics are associated with poor outcomes in these patients [3,23,24,25,26,27]. Similar attention is paid to peri-operative antibiotics [28]. However, there is a relative paucity of literature regarding the effect of delays in critical care medicine when the orders to execute are conditional on parameters defined by a provider in the ICU regarding the change in symptoms [17]. If the recommendations for medication engagement are not established for a long time, their implementation is juxtaposed with practitioner judgment [29,30,31,32,33,34]. This may create an awareness of ambiguity and less urgency, especially in the case of conditional orders, via perception bias [16,29]. ICU environments may be particularly sensitive to delays and secondary perception bias despite the intensive monitoring of patients. Furthermore, several medications have much less stringent dosing and application criteria [30,35,36,37]. For example, antipyretic medications used to treat fevers are deployed depending on specific indications [36,37]. Antihypertensive medications for controlling high blood pressure are implemented when criteria are met which are somewhat incoherently described in different illnesses [33,38]. Finally, the application of inhalers is guided by a set of symptoms that providers may interpret differently. Consequently, the person administrating these medications may perceive the application orders as much less urgent than antibiotics or other therapeutics [39]. However, research quantifying delays is difficult to conduct due to limited archival data that track patient outcomes in real-time and confounders introduced into any retrospective datasets [40,41].

Here, we focus on the effect of nursing shift changes on delays in care delivery by using this change as an external disruptor that is not driven by patient health but can affect the timely delivery of medication conditional on symptomatic treatment (antipyretics, vasodilators, and inhalers) in critical care settings [5,42]. During shift changes, care may be disrupted as several providers exchange information pertinent to a patient’s condition [17,43]. The disruption of care via medication delay is measured by administering medications commonly prescribed and administered as needed due to conditional orders. Here, we hypothesize that during shift changes, medication delays will increase as a result of the shift change and not provider-driven patient prioritization, allowing us to quantify the harm associated with such a delay.

## 2. Materials and Methods

### 2.1. Setting

A dataset extracted from the electronic medical records of an academic healthcare system in the northeast region of the United States covering four major hospitals was utilized here [35]. The critical care units of this academic healthcare system included medical, cardiac, surgical, neurological, and other focuses for adult patients. The data represent 100 critical care beds surveyed between January 2012 and December 2015.

There was no hospital-wide standardization of the hand-off between ICU nurses at shift change during data collection. Therefore, each unit typically utilizes its own methods for assigning patients and completing the hand-off. Most tend to arrive before 7:00 a.m. to receive a verbal description of the patient and their health status from the preceding nurse, followed by a description of physical findings. Once this process is complete, the incoming nurse is officially assigned to that patient.

### 2.2. Measuring Medication Delay

We focus on three medication groups (antipyretics, bronchodilators, and vasodilators) known to have benefits, known mechanisms of action, and which are available on the unit for rapid deployment (Table 1). The delay in their delivery also has a clear clinical effect. In addition, these medications are commonly prescribed to be administered as needed or conditionally upon admission to the ICU.

This study focuses on single-instance medication orders to be delivered to the patient as soon as possible if the conditions to administer them were met, per the standards of care. Therefore, we do not consider medications given on an ongoing basis (i.e., every 12 h). Contrary to this, if a patient is scheduled to receive medication at 8 a.m. and 8 p.m. every day and receives the medication at 8 a.m. and 7 p.m. instead, this early delivery (negative delay) could be detrimental to the quality of care. We also assume that when a physician orders a single-instance medication for immediate delivery, it represents the clinically appropriate moment that this medication should be administered. From this assumption, we infer that if the intent of the prescribing physician is immediate delivery, then any delay is detrimental to the quality of care.

To measure medication delay, our dataset archives the timestamps for when a medication was ordered and another timestamp when the healthcare provider administered the medication to the patient in the patient’s room. The moment a physician places an order for a medication, a timestamp is generated in the medication database for that patient and labeled the “medication order time”. After a nurse retrieves the medication and prepares it for delivery to the patient, the nurse administering the medication must scan the medication and then the patient’s wristband identification prior to actual medication delivery [27,44,45]. This safety protocol has the added benefit of accurately identifying the moment a medication was administered to the patient when the nurse scans the medication at the patient’s bedside. At this time, a second timestamp is created in the medication dataset labeled the “medication delivery time”. We define medication delay in this study as the difference (in minutes) between the “medication delivery time” and the “medication order time”. To accurately measure the impact of medication delays on patient health, we focus only on medications ordered to be delivered to the patient immediately and only once.

Vital readings in our dataset are electronically archived every 15 min. Seeing as each reading may be affected by unique interferences, we average the readings in the two hours before the patient received a medication order and in the two hours after the patient received it. If this two-hour average is within the ‘nominal’ vital range, we code the patient as being “healthy”. Otherwise, the patient is in an ‘unhealthy’ state. For the antipyretic group of medications, we tested the impact of antipyretic delays on the patient’s odds of entering a body temperature > 100 Fahrenheit [36,37]. For bronchodilators, we tested the odds that the patient enters a high respiratory rate state (readings above 20 breaths per minute). For vasodilators, we test the impact of delaying vasodilator administration on the odds that the patient develops a high blood pressure (mean arterial pressure (MAP) > 90 mmHg). These triggers were reported as accurate considering when the database was collected, the perception of trigger severity, and how quickly practice pattern changes [4,31,32,34,46,47].

### 2.3. Statistical Approach

Our first instrument is the nurse shift change. The nurse shift change occurs daily at 7 a.m. and 7 p.m., so, consequently, we identify this instrument during each instance of a medication order by using a binary indicator for whether or not the medication was ordered within the 6:30 a.m. to 7:30 a.m. or 6:30 p.m. to 7:30 p.m. time windows. Our second instrument, the average medication delay experienced by other patients on the same unit within the same hour, captures other exogenous adverse effects on the unit that affect medication delay. These adverse effects include, but are not limited to, census and high patient load effects, unit-level care coordination activities, and any external distractions between providers. The authors identified these instruments after personally observing activities on the unit during and outside shift change hours and confirmed by staff on follow-up. They are appropriate since they influence medication delivery, but are exogenous to patient health and can only influence patient health through their effects on medication delivery timeliness.

Regression estimation with instrumental variables occurs in two steps. In the first stage of our two-stage IV approach, we regress the confounding variable, medication delay, onto our instrumental variables, nursing shift change, and the average medication delay observed by other patients on the same unit within the same hour. To ensure we are conducting the appropriate risk-adjusted analysis, we include three groups of control variables in this first-stage regression model that may influence patient health and medication delay: (1) unit/hospital controls, (2) patient-medication level controls, and (3) time-related controls. These are outlined in Table 2. In the second stage, our dependent variable is a binary indicator representing whether the patient enters an adverse health state as measured by their average vital signs in the two hours following a medication order.

Using the two-hour vital average as our outcome, we include the same control variables from our first-stage model, with the key independent variable in this second-stage model being the exogenous medication delay obtained from the first stage. Model estimation was conducted using ordinary least squares (OLS) linear regression in the first stage and maximum likelihood estimation on binary-outcome logistic regression in the second stage. For comparison, we also conducted the naïve estimation of the second-stage outcome without our instrumental variables. All data cleaning and statistical analyses were completed using R [48].

## 3. Results

**Figure 1:**
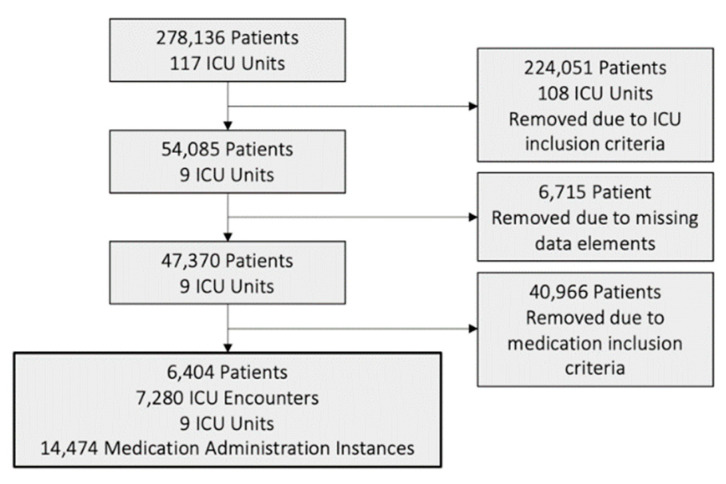
Flow chart detailing dataset inclusion criteria.

The records of a total of 47,370 patients were examined. Inclusion criteria are shown in Figure 1. From the original dataset, we selected a set of 9 ICU units that are comparable based on their ability to see medical/surgical patients. The other ICUs were either for subspecialties of patients requiring different care (for example, neuro and pediatric ICUs) or had a much smaller unit size, making it hard to compare across units. In addition, 6715 patients were removed from this original selection due to incomplete data in the cleaning process. A final sample of 6404 patients was selected due to the single-instance administration of antipyretics, vasodilators, or inhalers. The unit of analysis of our study is at the medication delivery level.

Patients in our final study sample have an average age of 61.4 ± 15.8 ($\bar{x}$ ± SD) and 43% of patients are female. The average ICU length of stay is 6 ± 7 days ($\bar{x}$ ± SD), with a median length of stay of 3.7 days (interquartile range of 1.9 days to 7.2 days). In addition, 59% of patient discharges from the hospital are routine and to the patient’s home (with follow-up home health care services), 33% of discharges are to a long-term care facility or nursing home, and 8% of patients expire during their time in the hospital (Table 3). Registered nurses (RNs) are typically assigned two patients per nurse, with the occasional 1:1 provider-to-patient ratio if particularly complex care is required. RNs are supported by one certified nursing assistant per eight patients, on average.

### 3.1. Estimated Effect of Nursing Shift Change on Medication Delay

**Figure 2:**
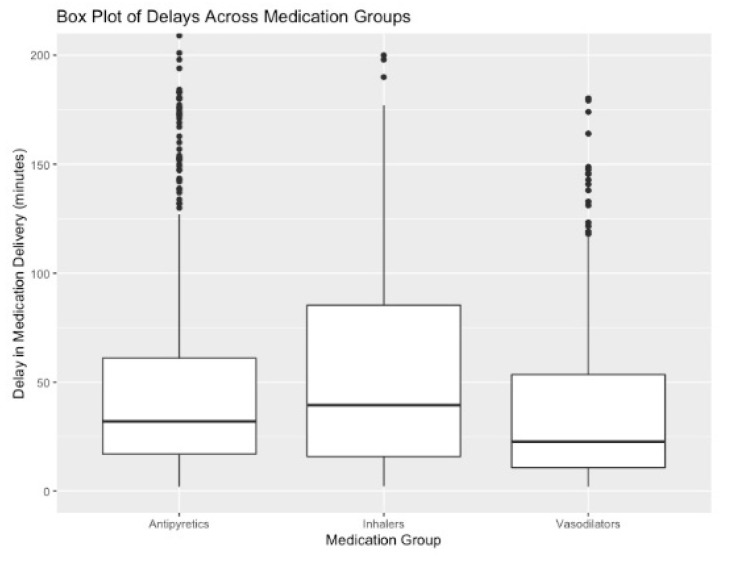
Box plots showing the distribution of observed delays across medication groups. The box plot shows the median and quartiles of observed delays (in minutes) of antipyretics, inhalers, and vasodilators. The mean delays (standard deviation) for these three groups are: 61.3 min (114 min) for antipyretics, 73.8 min (133 min) for inhalers, and 45.1 min (72 min) for vasodilators. This figure is truncated for clarity; the y-axis is truncated to show delays between 0 and 200 min.

The average delay across all medications included in our study is 88 min, with a median delay of 62 min (Figure 2). We found that medications ordered for immediate delivery during the time window around a nurse shift change have higher observed delays of 60.81 min (*p* < 0.001) on average for antipyretics, 39.51 min (*p* < 0.001) on average for bronchodilators, and 57.11 min (*p* < 0.001) on average for vasodilators. In addition, a one-minute increase in the average delay observed for all other patients on the same unit within the same hour of the medication order results in a 33.6 sec (*p* < 0.001) increase in the delay of antipyretics, a 45.6 s (*p* < 0.001) increase in the delay of inhalers, and a 12.6 s (*p* < 0.001) increase in the delay of vasodilators (Table 4).

### 3.2. Estimated Effect of Medication Delay on Patient Vital Status

Delaying antipyretics by one minute increased the patient’s odds of developing a fever in the two hours following the delay by 0.54% (*p* < 0.05; Table 5). Multiplying our finding by the observed empirical delay of antipyretics (61 min) translates to an increase in the odds that the patient experiences a high body temperature in the two hours following the medication order by 32.94% if antipyretics are delayed by 61 min.

Delaying bronchodilators by one minute increased the patient’s odds of entering a high respiratory state in the two hours following the delay by 1.06% (*p* < 0.05; Table 5). However, the naïve regression method found no significant impact of delaying inhalers on the odds that the patient entered a high respiratory rate state in the two hours following the delay. In other words, our results show that the observed average 75-min delay in inhalers seen in the data increases the odds that a patient experiences a high respiratory state in the two hours following the order by 79.5%.

Delaying vasodilators by one minute increased the odds that the patient entered a high blood pressure state in the two hours following the delay by 2.99% (*p* < 0.001, Table 5). Contrary to this, the naïve regression method demonstrated that the impact of delaying vasodilators on the odds that the patient entered a high blood pressure state is 1.05%. Using our finding and the observed empirical mean delay of vasodilators (45 min), we find that a 45-min delay of vasodilators increases the odds of hypertension in the two hours following the medication order by 134%.

## 4. Discussion

The objective of this study was to quantify the impact of delays in single, non-recurrent medication orders in the ICU for immediate delivery in case an order was prescribed as conditional on patient health status: more critical patients are often prioritized over less critical patients and receive their medications more quickly [28,29,49]. Delays in healthcare delivery are difficult to avoid, particularly in a setting as complex and fast-paced as the ICU [5,17,24,29]. We found that patients in the ICU experienced an average delay of 88 min for medications ordered to be administered right away. Our findings quantify a widely known phenomenon that has not been quantified to date—the interruption of care during shift changes for medications which are conditional for patient health vs. scheduled ones. In this paper, we focused our analysis on three medication groups of interest, (1) antipyretics, (2) bronchodilators, and (3) vasodilators, as well-established representatives of conditional medications [36,37]. Though quantifying the impact of these delays is difficult, similar data in sepsis demonstrated a significant increase in mortality [3,23]. The clinical assessment of the impact is likely to require much more granular data. Delays in delivering antipyretics may be related to delays in delivering antibiotics if the fever is driven by a septic process [3,23,39,50]. Delays in the administration of antibiotics have a direct translation to mortality. Delays in providing symptomatic treatment to lower blood pressure may increase cardiac events, while lack of timely delivery of inhalers increases the rate of intubation and mechanical ventilation [3,33,38,51].

Healthcare managers should devise strategies to mitigate some of the negative impacts of these delays on patient health. For example, vasodilators can be prioritized for patients recovering from heart surgery since their condition may signify a unique sensitivity to fluctuations in blood pressure. The intuitive way to improve healthcare delivery is to change the workflow in the hospital. However, not all intuitive solutions yield the desired outcomes [18,28,39,52]. Alternating nurse shift changes may help with continuity of care as adding an 11:00 a.m. to 11:00 p.m. shift eases the transition between 7:00 a.m. to 7:00 p.m. and 7:00 p.m. to 7:00 a.m. shifts. “Floating” nurses such as these could help balance the load of patients and coordinate care across shifts. Additionally, implementing a step in the ICU workflow dedicated to periodically assessing the medications prescribed as needed may also prevent the disruption of care caused by medication delays [53]. Critical analyses of truly beneficial treatments should also be conducted as focusing the work of healthcare staff on the most critical medical assignments is key to effective and efficient care as they may devise a solution that balances patient needs and the staff workflow. Implementing bundles is often seen to improve workflows, but this solution has mixed outcomes [50,52,54]. Increasing awareness of how medication type impacts delays is another interesting strategy [53]. Creating visual aids and a clear communication strategy helps deliver drugs, yet it is unclear if the delivery is timely [55,56]. Future research can combine our approach with predictive models to help providers better prioritize medication delivery to specific patients based on their health condition [57,58]. Beyond estimating the effect of medication delays for the average ICU patient, an even more powerful model could help providers identify the patient that would benefit the most from a certain medical intervention at any given time. Finally, implementing tele-ICU services as quality control may result in improved medication timing adherence, especially if additional assessment is needed before dispensing the conditional medications [42,59].

The strength of this study is our ability to leverage quasi-experimental methods to present causal estimates by identifying instrumental variables unique to the critical care setting. Given the confounding variable present in the dataset, it is not surprising that coefficients estimated using the naïve linear regression method are either insignificant or lower in magnitude than coefficients estimated using the IV method. A naïve regression without controlling for such unmeasured prioritization behavior (or confounding variables) would lead to biased results, suggesting that delays are less harmful than they are as the patients experiencing longer delays will tend to be less sick and therefore have better outcomes. To overcome this challenge, we used an instrumental variable approach to capture quasi-random medication delays across patients by selecting exogenous, instrumental variables independent of patient health status that capture increases in medication delays. The ideal instrument is related to the variable that suffers from unmeasured confounding (in this study, medication delays), but influences the outcomes of interest only through their effect on medication delays. Since this research question is difficult to study experimentally due to the ethical concerns surrounding intentionally delaying medications for a random subset of patients, we integrated a granular observational dataset with an instrumental variable (IV) estimation strategy to obtain unbiased estimates for the effect of delaying certain types of medications on patient health as measured through their vital signs. Since ICU care often aims to ensure that patients are spending time recovering from surgery or illness with healthy vital signs, our chosen health outcome captures the impact of medication delays on vital sign readings [4,5,16,17,18]. In addition, the medication groups included are commonly available on the unit, therefore greatly reducing the possibility that the observed delays are driven by retrieval from the pharmacy.

The presented study has limitations. Medication prescription patterns have evolved in last twenty years [3,36,37,38,47]. The collected database presents certain snapshots of practice at the time of data collection. Since this database was collected, several important changes have been introduced in healthcare, including protocolization, increased awareness of timely antibiotic administration, changes in practice patterns related to blood pressure goals, and treatment of fever [3,38]. Medications included here are mostly given as needed, and providers could specify different thresholds [5,18,39,60,61]. The staff may not be aware of the requirements for timely medication administration. Furthermore, some of the medications specified that they could be given in escalating or repetitive doses. This was not accounted for in this database. Limitations in this study also include electronically archived data and assumptions applied in the statistical modeling process. Here, because we do not observe a granular time series for the patient vital sign data, we aggregated the vital signs we did observe before and after medication delays to a two-hour window to minimize the impact of noisy measurements. We also assume in this study that medications within the same group have similar onset times and only consider single-instance non-recurrent medication orders. Future work can extend our analysis to a larger collection of medication groups, more specific medication delivery channels, and regularly scheduled medications.

Considering this study relies on a database created between 2011 and 2015, the data are not reflective of current workflow procedures in the ICU. This is particularly true of medication administration protocols, as a MAP of 90 mmHg or temperature of 100 F is no longer used to define dangerous health states for most patients or diagnoses. Although the thresholds for abnormal health states have been updated, the study’s principle remains relevant as major changes in health statuses may be observed following medication delays from workflow disruptions.

## 5. Conclusions

This study successfully quantified the impact of medication delays on patient health in the ICU during nursing shift changes. We demonstrated a significant disruption in care delivery during shift changes in providing symptomatic conditional treatments. The findings suggest that workflow optimization around shift change should be revised to minimize disruption.

## Figures and Tables

**Table 1:** Medication names included in each medication group.

Medication Group	Medication Names Included in Group
Antipyretics	Acetaminophen, Acetaminophen Suppository, Acetaminophen Oral Liquid, Acetaminophen Tablet.
Inhalers	Albuterol Inhaler, Albuterol-Ipratropium Inhaler, Ipratropium-Albuterol Nebulization, Beclomethasone 80 micrograms Inhaler, Ipratropium Inhaler, Levalbuterol 0.63 mg/3 mL Solution, Tiotropium Bromide.
Vasodilators	Hydralazine, Hydralazine Injection.

**Table 2:** Control variables included in regression models.

Control Group	Controls
Unit/Hospital Level	Hospital
	Unit
Patient/Medication Level	Patient Gender
	Patient Age
	Patient Complexity
	Primary Diagnosis Code
	Total ICU Stay Elapsed
	Pre-Order Health State
Time-Related Controls	Year Medication Ordered
	Month Medication Ordered
	Hour Medication Ordered

**Table 3:** Study sample summary statistics.

Measure	Summary Statistic (units)
Patient Age	61.4 ± 15.8 ($\bar{x}$ ± SD)
Patient Gender	43% Female
ICU Length of Stay	6 ± 7 days ($\bar{x}$ ± SD); median length of stay of 3.7 days (interquartile range of 1.9 days to 7.2 days)
Discharge Destinations	59% discharge to home; 33% discharge to a long-term care facility or nursing home; 8% of patients expire during the stay.

**Table 4:** Table of first-stage regression results for medication delays in antipyretics, inhalers, and vasodilators.

	Model (1) Antipyretic Delay	Model (2) Inhaler Delay	Model (3) Vasodilator Delay
Shift Change IV	60.81 *** (3.14)	39.51 *** (3.91)	57.11 *** (2.06)
Other Pt Delay IV (mins)	0.56 *** (0.01)	0.76 *** (0.02)	0.21 *** (0.01)
Pre-Vital Sign Controls	Yes	Yes	Yes
Unit Controls	Yes	Yes	Yes
Patient Controls	Yes	Yes	Yes
Time Controls	Yes	Yes	Yes
Observations	14,474	14,474	14,474
Adjusted R^2^	0.25	0.08	0.12
F-Statistic	1752.7 ***	1117.6 ***	1015.0 ***

*** *p* < 0.001. Note: F-Statistic and significance from ANOVA F-tests comparing models with our instruments included with models without instruments. The numbers represent the coefficient estimates; the numbers in parentheses represent standard errors on these estimates. Statistical significance is shown using asterisks.

**Table 5:** Table of second stage regression results with and without instrumental variables showing the impact of medication delays on the patient health states as measured by vital signs (for antipyretics, inhalers, and vasodilators).

	Antipyretic Delay High Temp (F)		Inhaler Delay High RR (Bpm)		Vasodilator Delay High MAP (mmHg)	
	Model (4)	Model (5)	Model (6)	Model (7)	Model (8)	Model (9)
Medication Delay (min)	0.54% * (0.22%)	0.10%(0.08%)	1.06% * (0.45%)	−0.05% (0.13%)	2.99% *** (0.70%)	1.05% *** (0.27%)
Year	−8.79% * (4.45%)	−9.14% * (4.44%)	−4.83% (2.78%)	−4.91% (2.78%)	1.61%(2.92%)	1.65%(2.91%)
Month	−2.44% * (1.22%)	−2.47% * (1.22%)	−1.22% (0.78%)	−1.16% (0.78%)	−0.05% (0.84%)	−0.06% (0.84%)
Hour	0.23%(0.49%)	0.29%(0.49%)	0.04%(0.33%)	0.05%(0.33%)	−0.90% * (0.37%)	−0.89% * (0.37%)
Gender: Male	26.64% ** (8.18%)	26.64% ** (8.17%)	−7.17% (5.05%)	−7.51% (5.04%)	19.07% ** (5.57%)	19.28% ** (5.57%)
Age	−1.00% *** (0.27%)	−1.00% *** (0.27%)	0.40% * (0.18%)	0.41% * (0.18%)	−0.72% *** (0.19%)	−0.72% *** (0.19%)
Comorbidities ^1^	1.03% ** (0.35%)	1.04% ** (0.35%)	1.16% *** (0.23%)	1.15% *** (0.23%)	−0.56% * (0.26%)	−0.57% * (0.26%)
IV Used	Yes	No	Yes	No	Yes	No
Naïve OLS	No	Yes	No	Yes	No	Yes

* *p* < 0.05; ** *p* < 0.01; *** *p* < 0.001 Coefficients represent the percent change of the odds that a patient enters an unhealthy vital sign health state as a result of a one minute delay in medication delivery from the medication group in consideration. The numbers represent the coefficient estimates; the numbers in parentheses represent standard errors on these estimates. Statistical significance is shown using asterisks. Row labels represent the variable being estimated. ^1^ Comorbidities are measured as a count variable of the number of unique diagnosis codes during the patient’s ICU stay.

## Data Availability

Data are available upon release by the IRB upon reasonable request.
